# Anti-bacterial activity of intermittent preventive treatment of malaria in pregnancy: comparative *in vitro *study of sulphadoxine-pyrimethamine, mefloquine, and azithromycin

**DOI:** 10.1186/1475-2875-9-303

**Published:** 2010-10-29

**Authors:** Mesküre Capan, Ghyslain Mombo-Ngoma, Athanasios Makristathis, Michael Ramharter

**Affiliations:** 1 Institute for Tropical Medicine, University of Tübingen, Tübingen, Germany; 2 Medical Research Unit, Albert Schweitzer Hospital, Lambaréné, Gabon; 3 Department of Parasitology and Mycology, University of Health Sciences, Libreville, Gabon; 4 Department of Laboratory Medicine, Division of Clinical Microbiology, Medical University of Vienna, Vienna, Austria; 5 Department of Medicine I, Division of Infectious Diseases and Tropical Medicine, Medical University of Vienna, Vienna, Austria

## Abstract

**Background:**

Intermittent preventive treatment of malaria with sulphadoxine-pyrimethamine (SP) is recommended for the prevention of malaria in pregnancy in sub-Saharan Africa. Increasing drug resistance necessitates the urgent evaluation of alternative drugs. Currently, the most promising candidates in clinical development are mefloquine and azithromycin. Besides the anti-malarial activity, SP is also a potent antibiotic and incurs significant anti-microbial activity when given as IPTp - though systematic clinical evaluation of this action is still lacking.

**Methods:**

In this study, the intrinsic anti-bacterial activity of mefloquine and azithromycin was assessed in comparison to sulphadoxine-pyrimethamine against bacterial pathogens with clinical importance in pregnancy in a standard microdilution assay.

**Results:**

SP was highly active against *Staphylococcus aureus *and *Streptococcus pneumoniae*. All tested Gram-positive bacteria, except *Enterococcus faecalis*, were sensitive to azithromycin. Additionally, azithromycin was active against *Neisseria gonorrhoeae*. Mefloquine showed good activity against pneumococci but lower *in vitro *action against all other tested pathogens.

**Conclusion:**

These data indicate important differences in the spectrum of anti-bacterial activity for the evaluated anti-malarial drugs. Given the large scale use of IPTp in Africa, the need for prospective clinical trials evaluating the impact of antibiotic activity of anti-malarials on maternal and foetal health and on the risk of promoting specific drug resistance of bacterial pathogens is discussed.

## Background

Malaria in pregnancy is associated with low birth-weight [1-3], pre-term delivery [4] and maternal anaemia [5] and is therefore an important cause of maternal, perinatal, and neonatal morbidity and mortality in pregnancy and the puerperium in sub-Saharan Africa [6,7]. The World Health Organization recommends intermittent preventive treatment of malaria in pregnancy with sulphadoxine-pyrimethamine (SP-IPTp) in order to reduce adverse health outcomes for pregnant women and their offspring [8,9]. Curative doses of SP are administered during routine antenatal visits at least twice after the first trimester in HIV negative and at least three times in HIV positive women. Due to rising drug resistance of *Plasmodium falciparum *against SP, potential alternative anti-malarial drugs have been proposed for future use as IPTp [10]. These compounds include amodiaquine, azithromycin, mefloquine, and combinations of these drugs with artemisinin derivatives or chloroquine [11,12].

Bacterial infections including sexually transmitted diseases, urinary tract infections, and group B streptococcal carriage are causes for considerable morbidity and mortality in pregnant women and the unborn child. In sub-Saharan Africa adequate diagnosis and treatment of these infections are often lacking. SP belongs to the class of anti-folates exerting considerable anti-microbial activity besides its anti-malarial activity. Anti-folate antibiotics show clinically important activity against *Streptococcus pneumoniae*, *Staphylococcus aureus*, *Escherichia coli*, and other pathogenic bacteria and are, therefore, used on a large scale for the treatment of urinary tract infections, skin and soft tissue infections, and in other indications [13,14]. Whereas mefloquine use is currently restricted to treating falciparum malaria, azithromycin is in use for the treatment of a variety of bacterial infections including respiratory tract infections and sexually transmitted diseases and it is under investigation for combination therapy of falciparum malaria [15,16].

IPTp with a drug exerting anti-bacterial activity may, therefore, offer a significant additional public health benefit by providing treatment for undetected or previously untreated bacterial infections in pregnant women. Conversely, widespread use of antibiotics may increase the risk for the development of drug resistance leading to future difficulties in the clinical management of bacterial infections.

This study aimed to assess the anti-bacterial activity of SP, mefloquine and azithromycin - the most promising candidate drugs for the replacement of SP for IPTp [15] - against common Gram-positive and Gram-negative bacteria *in vitro*.

## Methods

The minimal inhibitory concentrations (MIC) of mefloquine, azithromycin, and SP was assessed against bacterial pathogens with medical importance during pregnancy. For this purpose, common bacterial pathogens causing urinary tract infections (*Escherichia coli, Enterococcus faecalis)*, sexually transmitted diseases (*Neisseria gonorrhoeae*), skin and soft tissue infections (*Staphylococcus aureus*) and neonatal sepsis (Group B beta-haemolytic *streptococci; i.e. Streptococcus agalactiae*) were studied. Tested microorganisms consisted of clinical isolates and American Type Culture Collection (ATCC) strains as external controls. Bacteria were grown overnight at 37°C in Mueller Hinton broth with or without 2-5% horse-blood. *Neisseria gonorrhoeae *was incubated overnight at 35°C in an atmosphere containing 5% CO_2 _on New York City agar and chocolate agar.

All drugs were obtained from Sigma-Aldrich (Seelze, Germany) and were first dissolved and diluted to stock solutions. Further 1:2 dilutions of stock solutions were done with culture medium in order to achieve respective drug concentrations. Mueller Hinton broth medium was commercially prepared (Merck KGaA, Darmstadt, Germany). Fastidious broth medium was used for cultivation of *N. gonorrhoeae *and was prepared as described previously consisting of 35 g Columbia broth base, 5 g glucose, 5 g yeast extract, 2 g neopeptone, and 0.75 g agarose dissolved in 960 ml of distilled water [17]. A total of 30 ml haematin solution (0.05% [wt/vol] in 0.1 M NaOH) and 5 ml Tween 80 (10% [vol/vol]) was then added. The resultant broth was sterilized by autoclaving and 6 ml of pyridoxal solution (0.1% [wt/vol]) and 1.5 ml of NAD solution (1% wt/vol) were added.

### Determination of MICs

MICs were determined by employing a standard microdilution assay following Clinical and Laboratory Standards Institute (CLSI) guidelines with fastidious broth medium and Mueller Hinton broth and a bacterial turbidity of 0.5 McFarland. The final bacterial density was approximately 10^5 ^CFU/ml. The 96-well plates were incubated for 24 h at 35°C in a moist atmosphere containing 5% CO_2_. Positive control wells contained microorganisms without antibiotics. All tests were performed in duplicate and MICs were reported as arithmetic means.

### Classifiction of anti-bacterial activity

CLSI consensus cut off levels were used for the categorization of anti-bacterial activity. Azithromycin susceptibility was assessed using the following cut off levels for classification as sensitive, intermediate and resistant: *S. aureus *≤ 2 μg/ml, > 2 - < 8 μg/ml, ≥ 8 μg/ml; *S. pneumonia *and *S. agalactiae *≤ 0.5 μg/ml, > 0.5 - < 2 μg/ml, ≥ 2 μg/ml. In case of *N. gonorrhoeae*, due to a lack of CLSI recommendations, the European committee on anti-microbial susceptibility testing (EUCAST) definitions for azithromycin cut-off values (≤ 0.25 μg/ml; > 0.25 μg/ml - ≤ 0.5 μg/ml; > 0.5 μg/ml as sensitive, intermediate, resistant) were employed.

No break points are defined for the activity of azithromycin against *E. coli *and *Enterococcus *by CLSI and EUCAST. Cut off levels as proposed for *S. aureus *(≤ 2 μg/ml, > 2 - < 8 μg/ml, ≥ 8 μg/ml for susceptible, intermediate and resistant, respectively) were therefore used.

No recommendations are available for mefloquine and SP by CLSI or EUCAST and no previous publications on the interpretation of *in vitro *anti-bacterial activity of the two anti-malarial drugs were found. For the classification of SP activity - due to comparable anti-bacterial pharmacodynamics in the class of anti-folate antibiotics and similar molecular weights for trimethoprim and pyrimethamine (290 and 249, respectively) - CLSI and EUCAST threshold levels of anti-bacterial activity were used as defined for trimethoprim-sulphamethoxazole. These definitions are based on trimethoprim drug concentrations and were employed for pyrimethamine to classify SP activity. The CLSI cut off levels for classification as sensitive, intermediate and resistant are the following: *S. aureus *and *E.coli*: ≤ 2 μg/ml; > 2 - < 4 μg/ml; **≥ **4 μg/ml, *S. pneumonia*: ≤ 0.5 μg/ml; > 0.5 - < 4 μg/ml; **≥ **4 μg/ml. The EUCAST cut off levels: *S. agalactiae*: ≤ 1 μg/ml; > 1 - ≤ 2 μg/ml; > 2 μg/ml, *E. faecalis*: ˂ 0.03 μg/ml; ≥ 0.03 μg/ml - ≤ 1 μg/ml, > 1 μg/ml. For mefloquine the threshold of drug resistance was set considering available pharmacokinetic data in human patients and published *in vitro *inhibitory concentrations against *Plasmodium falciparum *[18-24]. Thus, the threshold for drug resistance was 0.265 μg/ml for mefloquine.

## Results

All bacterial isolates were sub-cultured after thawing prior to susceptibility assays (n = 34). Median MIC values of SP against Gram-positive bacteria were as follows: *S. aureus *16 μg/ml, *S. agalactiae *24 μg/ml, *S. pneumoniae *4 μg/ml, and *E. faecalis *12 μg/ml (Table 1, Table 2 and Additional File 1). Median MICs were considerably higher for *N. gonorrhoeae *and *E. coli *(256 μg/ml and 128 μg/ml, respectively). SP showed high or intermediate activity against all tested Gram-positive bacteria, whereas *E. coli *and *N. gonorrhoeae *were classified as resistant to SP.

**Table 1:** Median minimal inhibitory concentrations (MIC) of anti-malarials against selected Gram-positive and Gram-negative bacteria.

Microorganisms	Median Minimal Inhibitory Concentration (μg/ml)		
	Sulphadoxine/Pyrimethamine	Mefloquine	Azithromycin
S. aureus (n = 5)	16	16	0.5
S. agalactiae (n = 4)	24	16	0.06
S. pneumoniae (n = 5)	4	0.06	0.01
E. faecalis (n = 5)	12	16	4
N. gonorrhoeae (n = 10)	256	8	0.01
E. coli (n = 5)	128	128	4

Thresholds for drug resistance:

Mefloquine: sensitive: ≤ 0.265 μg/ml

Sulphadoxine/Pyrimethamine (threshold based on fractional pyrimethamine concentration in 1:20 combination): sensitive, intermediate, resistant

*S. aureus *and *E. coli*: ≤ 2 μg/ml; > 2 - < 4 μg/ml; ≥ 4 μg/ml,

*S. pneumonia*: ≤ 0.5 μg/ml; > 0.5 - < 4 μg/ml; ≥ 4 μg/ml,

*S. agalactiae*: ≤ 1 μg/ml; > 1 - ≤ 2 μg/ml; > 2 μg/ml,

*E. faecalis*: ˂ 0.03 μg/ml; ≥ 0.03 - ≤ 1 μg/ml; > 1 μg/ml.

Azithromycin: sensitive, intermediate, resistant

*S. aureus*; *E. coli *and *E. faecalis *≤ 2 μg/ml, > 2 - < 8 μg/ml, ≥ 8 μg/ml;

*S. pneumonia *and *S. agalactiae *≤ 0.5 μg/ml, > 0.5 - < 2 μg/ml, ≥ 2 μg/ml;

*N. gonorrhoeae *≤ 0.25 μg/ml; > 0.25 μg/ml - ≤ 0.5 μg/ml; > 0.5 μg/ml;

A total of 34 bacterial isolates were evaluated for their *in vitro *drug susceptibility against mefloquine (Table 1, Table 2 and Additional File 1). The observed MIC of mefloquine was 16 μg/ml in all tested *S. aureus *and *E. faecalis *isolates. Similarly growth of *S. agalactiae *was completely inhibited at 16 μg/ml except for one isolate with a MIC of 32 μg/ml. The MIC of mefloquine against pneumococci and *N. gonorrhoeae *varied between 0.03 - 0.06 μg/ml and 4 - 16 μg/ml, respectively. Based on the observed MIC values *S. pneumonia *was classified as sensitive, *S. aureus*, *S. agalactiae*, *E. faecalis*, and *N. gonorrhoeae*, and *E. coli *(Median MIC 128 μg/ml) as resistant to the *in vitro *activity of mefloquine.

**Table 2:** Summary of *in vitro *anti-bacterial activity of sulphadoxine-pyrimethamine, mefloquine, and azithromycin.

	Sulphadoxine/Pyrimethamine	Mefloquine	Azithromycin
***S. aureus***	**+**	**-**	**+**
***S. agalactiae***	**~**	**-**	**+**
***S. pneumoniae***	**+**	**+**	**+**
***E. faecalis***	**~**	**-**	**~/-**
***N. gonorrhoeae***	**-**	**-**	**+**
***E. coli***	**-**	**-**	**~**

- Insufficient *in vitro *activity; ~ intermediate *in vitro *activity; + good *in vitro *activity

Median MIC values of azithromycin were 0.5 μg/ml, 0.06 μg/ml, 0.01 μg/ml and 0.01 μg/ml against *S. aureus*, *S. agalactiae*, *S. pneumonia*, and *N. gonorrhoeae*, respectively (Table 1, Table 2 and Additional File 1). Activity against *E. coli *(median MIC 4 μg/ml) and *E. faecalis *(median MIC 4 mg/ml) was considerably weaker. *Neisseria gonorrhoeae *and all Gram-positive bacteria - except for *E. faecalis *-were classified as being sensitive to azithromycin. *Escherichia coli *strains showed intermediate drug susceptibility against azithromycin *in vitro*.

## Discussion

This *in vitro *drug susceptibility study showed a broad spectrum of anti-bacterial activity of SP against Gram-positive and low activity against Gram-negative bacteria. Among the two currently proposed alternative drugs for IPTp - mefloquine and azithromycin - the latter shows an even broader anti-bacterial spectrum of activity as SP with good activity against *N. gonorrhoeae*. Interestingly, previous reports indicate *in vitro *activity of mefloquine against *E. coli *[25,26]. The present study, however, demonstrated high activity of mefloquine against pneumococci and low activity against all other bacteria. Whether these findings similarly translate into clinically relevant in vivo activity of mefloquine needs further investigation since no validated resistance thresholds are available for mefloquine.

This evaluation of the anti-microbial activity of anti-malarials *in vitro *may provide the basis for further clinical evaluation. Based on our data a clinically important effect on concurrent infectious diseases in pregnant women may be anticipated for SP and azithromycin and to a lesser extent for mefloquine. Previous data show that bacterial infections including sexually transmitted diseases, pneumococcal infections, and *S. agalactiae *colonisation contribute significantly to adverse pregnancy outcome in sub-Saharan Africa [27]. Similarly, there is evidence for significant improvement of maternal and child health by routine administration of appropriate anti-microbial drugs during pregnancy [28]. Given that urinary and genital tract infections are an important cause for premature delivery, it may be speculated that the routine administration of SP and azithromycin for IPTp may confer a reduction in the rate of prematurity due to the antibiotic effect. Similarly the potential for eradication of vaginal *S. agalactiae *colonization by IPTp with SP or azithromycin might prevent cases of neonatal sepsis. However, caution must be employed by the extrapolation of data on *in vitro *activity of drugs to anticipated *in vivo *efficacy. Clinical efficacy will ultimately depend - besides the intrinsic anti-bacterial activity as assessed in this study - on drug absorption, drug concentrations at the target sites, half-lives of drugs, and the local pattern of drug resistant pathogens. Additionally a limitation of our study lies in the absence of validated thresholds for the *in vitro *activity of SP and mefloquine. The proposed levels are extrapolated from antifolate antibiotics or based on thresholds of *in vitro *activity against *P. falciparum *and need further clinical validation [17].

The next generation of IPTp drugs will be chosen based on pharmacodynamic properties and its safety, tolerability, simplicity of administration, and cost. Based on the hypothesis of a collateral health benefit by the administration of anti-malarials with activity against relevant bacterial pathogens, it may seem desirable to choose the next IPTp drug based on both its anti-malarial and anti-bacterial pharmacodynamic properties. Whether such an approach is justified or not is however to date unknown. Whereas it may look attractive to simultaneously treat concomitant and potentially deleterious bacterial infections by routine administration of anti-malarials, this strategy may also prove hazardous. Large-scale use of drugs with anti-bacterial activity may speed up the process of selection of drug resistant bacterial isolates. Interestingly, there is evidence for the development of antibiotic drug resistance by cross-resistance with anti-malarial drugs [29]. Epidemiologic evidence linking the development of quinolone resistant Gram-negative bacteria with large-scale use of chloroquine and the recent development and spread of quinolone resistant *N. gonorrhoeae *strains are illustrative examples for this phenomenon [30]. In this context the potential selection of drug resistance against anti-folate and macrolide antibiotics by the use of SP and azithromycin as IPTp is of particular concern. The threat of promoting drug resistance against commonly used antibiotics is particularly worrying for sub-Saharan Africa where microbiologic analysis of infections is rarely performed and alternative antibiotics for drug resistant pathogens are often not affordable.

## Conclusion

These data indicate that sulphadoxine-pyrimethamine and azithromycin are active against a broad spectrum of bacterial pathogens whereas mefloquine's activity is restricted to pneumococci. Whether the choice of a second generation IPTp drug with broad or narrow anti-bacterial spectrum is favourable for maternal and foetal health, is currently unknown. Further clinical trials evaluating the efficacy of IPTp against concomitant bacterial infections and the impact of their large scale use on the development and spread of antibiotic drug resistance are therefore necessary to allow an informed decision on the next IPTp drug for Africa.

## Competing interests

The authors declare that they have no competing interests.

## Authors' contributions

MR and GMN developed the concept and design of the study, were responsible for data analysis, and drafting of the manuscript. MC participated in study design, was responsible for the collection of data, and contributed to data analysis, interpretation, and drafting of the manuscript. AM participated in study design, data analysis, and critical review of the manuscript. All authors read and approved the final manuscript.

## Supplementary Material

Additional file 1 **Minimal inhibitory concentrations (MIC) of anti-malarials against selected Gram positive and Gram negative bacteria**. Listing of minimal inhibitory concentrations of all isolates. Click here for file
