# Supplementary material for: Anti-bacterial activity of intermittent preventive treatment of malaria in pregnancy: comparative in vitro study of sulphadoxine-pyrimethamine, mefloquine, and azithromycin
Source: Malar J. 2010 Oct 29;9:303. doi: 10.1186/1475-2875-9-303 (PMC2984572; doi:10.1186/1475-2875-9-303)
Supplement: Additional file 1 — Minimal inhibitory concentrations (MIC) of anti-malarials against selected Gram positive and Gram negative bacteria. Listing of minimal inhibitory concentrations of all isolates. [file 1475-2875-9-303-S1.DOC]

**Additional File 1.**

Minimal inhibitory concentrations (MIC) of anti-malarials against selected Gram positive and Gram negative bacteria.

| **Microorganisms**  **(ATCC & N° of isolate)** | **Minimal Inhibitory Concentration (µg/ml)** | | |
| --- | --- | --- | --- |
|  | **Sulphadoxine/Pyrimethamine** | **Mefloquine** | **Azithromycin** |
| **S. aureus** |  |  |  |
| ATCC25923 | 16 | 16 | 0.25 |
| 1 | 16 | 16 | 0.75 |
| 2 | 16 | 16 | 0.5 |
| 3 | 8 | 16 | 0.5 |
| 4 | 32 | 16 | 0.5 |
| **Median MICs** | **16** | **16** | **0.5** |
| **S. agalactiae** |  |  |  |
| 1 | 32 | 32 | 0.06 |
| 2 | 16 | 16 | 0.06 |
| 3 | 16 | 16 | 256 |
| 4 | 128 | 16 | 0.06 |
| **Median MICs** | **24** | **16** | **0.06** |
| **S. pneumoniae** |  |  |  |
| ATCC 49619 | 4 | 0.03 | 0.25 |
| 1 | 1.5 | 0.03 | 0.01 |
| 2 | 8 | 0.06 | 0.01 |
| 3 | 4 | 0.06 | 0.01 |
| 4 | 4 | 0.06 | 1 |
| **Median MICs** | **4** | **0.06** | **0.01** |
| **E. faecalis** |  |  |  |
| ATCC 29212 | 2 | 16 | 3 |
| 1 | 12 | 16 | 4 |
| 2 | 12 | 16 | 256 |
| 3 | 16 | 16 | 1 |
| 4 | 8 | 16 | 4 |
| **Median MICs** | **12** | **16** | **4** |
| **N. gonorrhoeae** |  |  |  |
| ATCC 43069 | 256 | 8 | 0.01 |
| 1 | 128 | 5 | 0.01 |
| 2 | 256 | 4 | 0.01 |
| 3 | 256 | 8 | 0.125 |
| 4 | 256 | 16 | 0.125 |
| 5 | 256 | 8 | 0.01 |
| 6 | 128 | 8 | 0.01 |
| 7 | 64 | 8 | 0.125 |
| 8 | 256 | 16 | 0.01 |
| 9 | 256 | 16 | 0.01 |
| **Median MICs** | **256** | **8** | **0.01** |
| **E. coli** |  |  |  |
| ATCC 25922 | 192 | 64 | 4 |
| 1 | 128 | 128 | 4 |
| 2 | 128 | 128 | 2 |
| 3 | 128 | 256 | 4 |
| 4 | 256 | 128 | 4 |
| **MEDIAN MICs** | **128** | **128** | **4** |

**Thresholds for drug resistance:**

Mefloquine: sensitive ≤ 0.265 µg/ml

Sulphadoxine/Pyrimethamine: (threshold based on fractional pyrimethamine concentration in 1:20 combination): sensitive, intermediate, resistant

*S. aureus* and *E. coli*: ≤ 2 µg/ml; > 2 - < 4 µg/ml; ≥ 4 µg/ml,

*S. pneumonia*: ≤ 0.5 µg/ml; > 0.5 - < 4 µg/ml; ≥ 4 µg/ml,

*S. agalactiae*: ≤ 1 µg/ml; > 1 - ≤ 2 µg/ml; > 2 µg/ml,

*E. faecalis*: ˂ 0.03 µg/ml; ≥ 0.03 - ≤ 1 µg/ml; > 1 µg/ml.

Azithromycin: sensitive, intermediate, resistant

*S. aureus*; *E. coli* and *E. faecalis* ≤ 2 µg/ml, > 2 – < 8 µg/ml, ≥ 8 µg/ml;

*S. pneumonia* and *S. agalactiae* ≤ 0.5 µg/ml, > 0.5 – < 2 µg/ml, ≥ 2 µg/ml;

*N. gonorrhoeae* ≤ 0.25 µg/ml; > 0.25 µg/ml - ≤ 0.5 µg/ml; > 0.5 µg/ml;

ATCC: American Type Culture Collection
